# Factors That Most Influence the Choice for Fast Food in a Sample of Higher Education Students in Portugal

**DOI:** 10.3390/nu16071007

**Published:** 2024-03-29

**Authors:** Leandro Oliveira, António Raposo

**Affiliations:** 1 CBIOS (Research Center for Biosciences and Health Technologies), Universidade Lusófona de Humanidades e Tecnologias, Campo Grande 376, 1749-024 Lisboa, Portugal; 2 Polytechnic Institute of Coimbra, Coimbra Health School, Rua 5 de Outubro—S. Martinho do Bispo, Apartado 7006, 3046-854 Coimbra, Portugal

**Keywords:** fast food, food choice, higher education, students, university students

## Abstract

The frequency of fast food consumption among higher education students is high, causing worrying implications for public health. This study aims to relate the factors that influence the choice for fast food with social factors, nutritional status, and fast food consumption in a sample of higher education students in Portugal. An online questionnaire was developed and disseminated by social networks among students during the first half of 2023. Two hundred and thirty-seven students participated, mainly female (65.4%), who were attending public higher education institutions (59.1%), with a median of age of 20.0 (19.0; 22.0) years, and about 20% of the sample had overweight. Approximately 80% consumed fast food, and almost 40% consumed it more than once or twice a week. Predominantly (78.0%), they chose hamburger meals, spending EUR 8.0 per meal. The factors that most influenced the choice of fast food were ease or convenience of preparation (59.9%), price (48.5%), and flavor (28.3%). There were also differences between sexes and between those attending public and private higher education institutions regarding whether they usually consume fast food or not. The body mass index was positively associated with age (r: 0.142; *p* = 0.029) and with fast food spending (r: 0.146; *p* = 0.024). The results have implications for public health and clinical nutrition, and can support more effective strategies to improve food choices in higher education students.

## 1. Introduction

Ultra-processed foods are composed of ingredients mainly intended for industrial use, which originate from multiple manufacturing procedures (thus, “ultra-processed”), several of which require technologically advanced equipment and methods (for example, sugary and salty snacks, reconstituted meats, hamburgers, pizzas and confectionery products, nuggets, etc.) [1]. Thus, it appears that a fast food meal is composed of ultra-processed foods [1]. Fast food meals are characterized by their prompt availability, simplified preparation process, and emphasis on efficiency, allowing customers to receive their food in a matter of minutes after making the order [2].

Fast food is often associated with restaurant chains that offer a limited menu of standard options, such as hamburgers, chips, pizza, sandwiches, sodas, and other foods that are prepared quickly and intended for immediate consumption [3,4]. These establishments are known for their casual atmosphere and their facilities often designed to accommodate clients who want quick meals, whether on site, during travel, or through delivery services [4,5].

Both the fast food industry and the consumption of ultra-processed foods and fast food meals have increased dramatically around the world [5,6,7,8,9]. Ingestion of ultra-processed foods increased in the adult population of the United States from 2001–2002 to 2017–2018 (from 53.5% to 57.0% of calories consumed) [8]. In the European context, it was found that the energy contribution of ultra-processed products, including drinks, had notable variations, ranging from 14% to 44%. Lower proportions were observed in Italy and Romania, while the highest were identified in the United Kingdom and Sweden. In the case of Finland, Spain, and the United Kingdom, increases in consumption of these types of food and beverage were recorded, ranging from 3% to 9% [9]. In Portugal, according to Marktest’s TGI study for the year 2021, approximately 4,384,000 individuals said that they had consumed fast food meals in restaurants in the last 12 months, which is about 51.2% of residents of the Portuguese mainland aged 15 years or over [10]. Regarding higher education students, there has been a significant frequency of fast food consumption. These values can range from about three times a month [11] to two or more times a week [12,13].

Different factors have shown significant influences on frequent fast food consumption, including characteristics such as sex, young age group, higher socioeconomic status [11,14,15], and higher body mass index (indicating pre-obesity or obesity) [16,17]. Factors such as taste, brand reputation, ease of access, convenient location, price, promotional offers, and fast service play significant roles in increasing fast food consumption in higher education students [14].

Fast food consumption is a public health concern because it is associated with an unhealthy diet [5], as well as the risk of certain chronic diseases such as obesity [5,16,18,19,20], cardiovascular diseases [19,20], and diabetes [19,20], among others [20]. 

Higher education students are a particularly vulnerable group, as they are in a phase of life marked by various transformations, which include spending long periods away from home and staying at university campuses. This transition exerts a significant influence on eating patterns, often resulting in unwanted increases in weight [15]. This may have significant implications in the future, as weight gain during the young adult years is recognized as a significant risk factor for the development of obesity in the late phase of adulthood [21].

In this way, the purpose of this study is to identify and relate the factors that influence fast food consumption, considering social factors and nutritional status, in a sample of higher education students in Portugal. Understanding these factors will allow for the creation of more effective approaches to promoting healthier and more sustainable food choices in society.

## 2. Materials and Methods

### 2.1. Study Design and Data Collection

This study had a cross-sectional design and selected higher education students in Portugal as its target population. The inclusion criteria were being a higher education student in Portugal and being 18 years old or older; the exclusion criteria were not being a higher education student and being under 18 years old. For data collection, an online questionnaire built on the Google Forms^®^ platform was used, and all data were self-reported by participants. The questionnaire was disseminated during the first half of 2023 by the students’ social networks. They were asked to share by other colleagues, making the process a probabilistic sampling of the snowball type.

The questionnaire addressed different aspects, including sociodemographic characteristics and fast food consumption. These areas encompassed information such as sex, age, type of institution attended, anthropometric data, consumption patterns, and fast food frequency. In addition, the questionnaire explored the factors that exert the most influence on the choice of fast food and requested an estimate of the average value (EUR) paid per meal of this type.

The calculation of body mass index was performed using the weight (kg)/height^2^ (m) [22] formula. The results were then categorized following the criteria established by the World Health Organization for adults [23].

The factors that influence food choice were evaluated through a list of items obtained from a previous study [24]. As a result, the following determinants influencing food selection were chosen and adapted: F1. I don’t know; F2. Habit or routine; F3. The taste of food; F4. Food price; F5. Managing weight; F6. Food availability; F7. Presentation or packaging; F8. Another person decides most of the foods that I eat; F9. Vegetarian food or other special habits; F10. Content of additives, dyes, and preservatives; F11. My cultural, religious, or ethnic roots; F12. Ease or convenience of preparation; F13. Trying to have a healthy diet; and F14. Quality or freshness of food. Additionally, the “other” option was included, in which participants were requested to provide additional specifications. These answers were later grouped to form other variables: F16. Other—Socializing with friends/family; F17. Other—Lack of desire to cook; F18. Other—Quantity/size portion of meal; F19. Other—Craving or desire for fast food.

### 2.2. Ethical Approval

This study was conducted in accordance with the ethical principles stipulated in the 1964 Helsinki Declaration and its subsequent amendments, as well as in accordance with comparable ethical norms. Informed consent was obtained, and the study’s procedures and objectives were explained in detail. Approval was obtained for the study by the Ethics Commission of the School of Health Sciences and Technologies of the Lusófona University (P03-23).

### 2.3. Data Processing and Analysis

The IBM SPSS Statistics software, version 26 for Windows, was used to perform the statistical analyses in this study. For the statistical description, the mean and standard deviation (SD) were calculated, in addition to the absolute (n) and relative (%) frequencies. To investigate the relationship between the variables, the chi-square test or the exact test of Fisher was used (as appropriate) to evaluate the independence between variable pairs. To compare the ordained averages between independent groups, the Mann–Whitney test was used. In addition, the Pearson correlation coefficient (*r*) was used to analyze the association between continuous variables. The null hypothesis was rejected when *p* < 0.05, indicating statistical significance.

For statistical analysis, the type of educational institution was grouped into public or private, and the public non-state option was added to the private due to its similarities. The variables related to the factors influencing the choice of fast food were treated to present the absolute and relative frequencies related to participants who chose or did not choose a certain factor, transforming it into a dichotomous variable (yes/no).

## 3. Results

This study included 237 participants, most of them female (65.4%), attending public higher education institutions (59.1%), with an age of 21.1 (SD: 5.0) years (Table 1). Most had a normal weight (75.1%); however, 21.4% had overweight. About 80% of participants used to consume fast food, and about half reported a frequency of consumption of two to three times a month. Most participants (78.0%) used to opt for a hamburger meal and used to spend EUR 8.6 (SD: 3.0) on each meal. Regarding the comparison between sexes, it was found that the males had higher body mass index values (*p* < 0.001), a higher prevalence of overweight (*p* = 0.015), a higher prevalence of fast food consumption (*p* = 0.002), and a greater frequency of its consumption (*p* = 0.034) in relation to females.

Table 2 presents the relationships between the factors that exert the most influence on the choice of fast food, i.e., sex, type of educational establishment, fast food consumption, body mass index (kg/m^2^), and amount spent on each fast food meal, among university students. The three factors most pointed out by higher education students as those which most influenced their fast food consumption were ease or convenience of preparation (59.9%), price (48.5%), and flavor (28.3%). On the other hand, the least mentioned factors, besides “I don’t know” (97.9%), were quantity/size portion of the meal (99.6); vegetarian feeding or other special habits (98.7%); cultural, religious, or ethnic roots (98.7%); and another person decides most of the foods that I eat (98.3%).

A significantly higher proportion of male participants pointed to the desire to consume fast food as the main factor influencing the choice for fast food compared to female participants (*p* = 0.011). A higher proportion of individuals who attended private education mentioned that living with friends/family had a greater influence on the choice of fast food compared to public education students (*p* = 0.017). A significantly higher proportion of individuals who did not have the habit of consuming fast food mentioned that the factors of trying to have a healthy diet (*p* = 0.010) and controlling weight (*p* = 0.002) had a greater influence on the choice of fast food compared to those who admitted to the habit of consuming fast food. In addition, individuals who mentioned the factor of controlling weight as influencing their choice by fast food had higher body mass indices than their counterparts (*p* = 0.006). On the other hand, individuals who indicated the will or desire to consume fast food as an influencing factor in their choice had lower body mass indices than their peers (*p* = 0.001). Individuals who mentioned that they did not know which factors influenced the choice for fast food had a higher median value paid for each fast food meal in relation to their counterparts (*p* = 0.045). Finally, those who mentioned the price factor (*p* < 0.001) and food availability (*p* = 0.038) as influences in their choices for fast food presented a smaller median value paid for each fast food meal compared to their counterparts. There was no relationship between age and the factors that most influenced the choice of fast food among higher education students.

Correlation was also tested for age, body mass index, and the amount spent on the fast food meal. It was found that when the age was higher, the body mass index was also higher (*r*: 0.142; *p* = 0.029). When the body mass index was higher, the amount spent on fast food meals was also higher (R: 0.146; *p* = 0.024). No relationship was found between age and the amount spent on fast food meals.

## 4. Discussion

This is a cross-sectional study that involves students from higher education in Portugal, and whose purpose is to recognize and establish relationships between the factors that influence the decision to choose fast food, considering social factors, nutritional status, and fast food consumption.

In this study, about 75.1% of participants were considered normal. However, 21.4% of the sample had overweight. This suggests that an important proportion of participants was overweight. In fact, this overweight value meets that found in other studies of higher education students, pointing to a prevalence of 20% [16,17,25]. However, this is lower than other studies which have pointed to a prevalence of overweight of around 30%, such as studies of higher education students in Pakistan [26] and Peru [18], and a prevalence of up to 40% has been found in Jordan [27].

In fact, approximately 90% of participants reported consuming fast food, half of which mentioned consuming fast food two to three times a month, but almost 35% reported a consumption of one to six times a week. This indicates that fast food was relatively popular with the participants, with a moderate frequency of consumption. The vast majority of participants (78.0%) used to opt for a hamburger meal when consuming fast food, and spent about EUR 8 on each meal.

According to a study that took place in Viseu (Portugal) with a sample of 150 adults, it was reported that much of the inquired population does not consider themselves consumers of fast food (77.3%), often claiming consuming balanced meals (83%) [28]. Thus, this is practically the opposite of what was observed in the students of higher education in the present study. However, a more recent study [10] pointed to a prevalence of about 50% of fast food consumption in adults, even lower than that found in the students. Regarding the frequency of consumption, a study [13] in Jordan reported fast food consumption equal to or greater than once a week for 59.4% of students; another at the University of Aleppo pointed out that about 70% eat fast food once a week or more [16]. This may be due to the extensive workload at the college in addition to everyday tiredness, which may contribute to fast food becoming a popular option among students. Also, as most fast food meals do not have nutritional labeling, or this is not easily available/visible to consumers, it can lead to a real notion of the energy they are consuming. Thus, there may be an unrestricted consumption (from the conscious point of view) of this type of foods, which are highly palatable, but also energetic and rich in fats, simple sugars, and salt [29,30]. In fact, according to a study in Aleppo, higher education students requested meals with significantly lower energy values when opting for menu items with nutritional information compared to menus without this information [31]. Another more recent study showed that individuals who received direct information about healthy options present on fast food menus showed a greater tendency to select healthier food products compared to those exposed to more discreet integrated health information [32].

Regarding the amount spent, it is similar to the amount paid in a canteen of a private university, but much higher than the price paid for a complete meal in a social canteen associated with a public university, which is around EUR 2.8 [33]. Thus, encouragement to choose the school canteen to make their meals can bring not only economic benefits, but the health of the students [34]. 

The three main factors most pointed out by higher education students as influences on their fast food consumption are the ease/convenience of preparation, price, and taste. This suggests that practicality and cost are significant considerations in deciding what to eat [14]. The taste is also an important factor, which highlights the importance of palatability in food choice [1,12,14]. The discrepancy in body mass indices between the sexes suggests that men have, on average, higher rates compared to women, possibly related to more frequent fast food consumption by men [11]. In addition, the difference between the sexes in relation to the desire/willingness to consume fast food as an influencing factor is interesting. It indicates that men can be more driven by desire when choosing fast food, so they also pay more for each fast food meal, while women may consider other more influential factors in their choices [11]. For example, female students tend to demonstrate a higher level of concern for food and weight compared to males [35]. 

The association between private school students and the influence of living with friends/family on the choice of fast food suggests that social and group factors may be more relevant to this specific group. This may be related to differences in the social environments between the two teaching groups [36]. 

The absence of a habit of consuming fast food is related to a greater tendency of seeking healthy eating and weight control, indicating awareness of food and health choices. The association between factors that influence the choice of fast food and body mass index reveals various approaches to weight management and food choices. Those motivated by the desire to consume fast food had lower body mass indices, while those who prioritized weight control showed higher indices. In fact, it can be thought that those who have lower body mass indices may not have as much concern for weight control and may consume fast food with lower restrictions; the intention to consume fast food is a predictor of its consumption [11]. On the other hand, individuals with higher body mass indices give priority to controlling their weight. Knowing that fast food meals have high caloric value and are rich in fats, sugars, and salt, i.e., they are not healthy and have implications for weight gain [1,19], they may tend to avoid these kinds of meals.

### 4.1. Implications for Public Health and Clinical Nutrition

The implications of the presented results in terms of public health and clinical nutrition are significant and can help guide policies and interventions to improve the health of higher education students and promote healthier food choices. The results point out that factors such as ease, price, and taste influence food choices. Therefore, public health strategies should focus on making healthy options more accessible, convenient, and tasty in order to encourage students to choose nutritious meals [36]. The impact of living with friends/family on fast food choices suggests that social influence plays an important role. Awareness programs can emphasize how food choices can be influenced by the social environment and promote healthier eating habits in social contexts.

In addition, the relationship between influencing factors and body mass index suggests that clinical nutrition interventions can benefit from being personalized according to individual motivations. Health professionals can adapt their approaches based on the main factors that influence each patient. Given that frequent consumption of highly processed foods (such as fast food) is associated with health risks, such as obesity and cardiovascular disease [19], interventions based on these results can help to prevent these conditions.

Thus, the results found herein have important implications for both public health promotion and clinical nutrition. Understanding the factors that influence food choices allows for the creation of more effective strategies to improve the population’s eating habits and address food-related health issues.

### 4.2. Limitations and Future Directions

This investigation has some limitations that should be considered when interpreting their results and generalizing the conclusions. As this is a cross-sectional study, the identified correlations do not indicate direct causality. While the sample size is adequate, it is crucial that it accurately reflects the demographic composition of students to ensure the validity of the study’s findings. Inadequate representation of this population group may limit the generalizability of the results and lead to biased conclusions. Therefore, efforts should be made in future studies to ensure that the sample includes diverse individuals with varying characteristics, backgrounds, and experiences typical of students. By achieving more comprehensive representation, researchers can enhance the robustness and reliability of the study’s outcomes, allowing for a more accurate generalization of the results to the broader population of Portuguese higher education students. Other factors not measured may be contributing to the associations found, for example, the food environment of higher education institutions that students attend and the existence or nonexistence of a university canteen. In addition, the data collection period was quite extended, coinciding with the beginning of the school semester, and it is known that behavioral and health trends can diverge over time, so the results may be specific to the period of data collection, and what may be relevant at one point may not be at another. The fact that the data were self-related, especially weight and height, may have been the subject of a sub/on estimation, which would have implications for the results of the nutritional status classification.

Studies on factors that influence fast food choice, their impacts on health, and food behavior, higher education students are fundamental to understanding and addressing challenges related to healthy eating and public health. In the future, studies are suggested to evaluate the effectiveness of educational interventions, awareness campaigns, and public policies designed to reduce fast food consumption and promote healthier food choices in higher education students. Other studies that more deeply analyze the psychological, social, and economic factors that influence fast food choices, including the influence of advertising, social media, and the consumer environment, should also be conducted.

## 5. Conclusions

About 21.4% of the participants were overweight. Moreover, around 90% of the participants acknowledged consuming fast food, with half of them doing so two to three times a month, while 34.2% did so more than once a week, and 2.5% consumed it once or more times a day. Most participants preferred hamburgers when they consumed fast food and spent, on average, about EUR 8 on each meal of this type. The main factors that influenced fast food consumption among higher education students included convenience, price, and taste. In addition, significant differences were observed based on sex, the type of higher education institution (public or private), and the frequency of fast food consumption. A positive association was also found between body mass index and age, as well as between body mass index and fast food spending. The results of this research have important implications for public health and clinical nutrition, providing insights that can be used to develop more effective strategies to improve the food choices of higher education students.

## Figures and Tables

**Table 1:** Personal characteristics, fast food consumption of participants, and the differences between sexes (n = 237).

		Sex		
	Total n (%)	Male n (%)	Female n (%)	*p*
Total	237 (100.0)	82 (34.6)	155 (65.4)	----
Type of educational institution				
Public	140 (59.1)	52 (63.4)	88 (56.8)	0.335 ^a^
Public—non-state	8 (3.4)	3 (3.7)	5 (3.2)	
Private	89 (37.6)	27 (32.9)	62 (40.0)	
Nutritional status				
Low weight	8 (3.4)	3 (3.7)	5 (3.2)	0.015 *^b^
Normal weight	178 (75.1)	52 (63.4)	126 (81.3)	
Pre-obesity	41 (17.3)	22 (26.8)	19 (12.3)	
Obesity	10 (4.2)	5 (6.1)	5 (3.2)	
Do you usually consume fast food?				
Yes	181 (76.4)	53 (64.6)	128 (82.6)	0.002 *^a^
No	56 (23.6)	29 (35.4)	22 (17.4)	
How often do you consume fast food?				
Never or less than once a month	31 (13.1)	12 (14.6)	19 (12.3)	0.034 *^b^
Two to three times a month	119 (50.2)	31 (37.8)	88 (56.8)	
One to two times a week	61 (25.7)	28 (34.1)	33 (21.3)	
Three to four times a week	16 (6.8)	5 (6.1)	11 (7.1)	
Five to six times a week	4 (1.7)	3 (3.7)	1 (0.6)	
Daily	5 (2.1)	3 (3.7)	2 (1.3)	
Two to three times a day	1 (0.4)	0 (0.0)	1 (0.6)	
What kind of meal is it?				
Hamburger	185 (78.0)	66 (80.5)	119 (76.8)	0.652 ^a^
Pizza	30 (12.7)	8 (9.8)	22 (14.2)	
Other	22 (9.3)	8 (9.8)	14 (9.0)	
	Mean (SD)	Mean (SD)	Mean (SD)	*p*
Age (years)	21.1 (5.0)	21.5 (4.9)	20.9 (5.1)	0.060 ^c^
Body mass index (kg/m^2^)	23.0 (3.5)	24.0 (4.0)	22.4 (3.0)	<0.001 *^c^
Amount spent on each fast food meal (€)	8.6 (3.0)	9.0 (3.0)	8.4 (2.9)	0.070 ^c^

* p < 0.005; ^a^ Chi-squared test; ^b^ Fisher’s exact test; ^c^ Mann–Whitney test.

**Table 2:** Relationship between factors that have the most influence on choice of fast food, i.e., sex, type of educational establishment, consumption of fast food, body mass index (kg/m^2^), and amount spent on each fast food meal, among higher education students.

Factors That Have the Most Influence on Your Choice for Fast Food:	No/ Yes	Total n (%)	Sex			Type of Educational Establishment		
			Male n (%)	Femalen (%)	*p*	Publicn (%)	Private n (%)	*p*
F1. I don’t know	No	232 (97.9)	80 (97.6)	152 (98.1)	1.000 ^a^	136 (97.1)	96 (99.0)	0.651 ^a^
	Yes	5 (2.1)	2 (2.4)	3 (1.9)		4 (2.9)	1 (1.0)	
F2. Habit or routine	No	200 (84.4)	65 (79.3)	135 (87.1)	0.114 ^b^	119 (85.0)	81 (83.5)	0.755 ^b^
	Yes	37 (15.6)	17 (20.7)	20 (12.9)		21 (15.0)	16 (16.5)	
F3. The taste of food	No	170 (71.7)	56 (68.3)	114 (73.5)	0.393 ^b^	104 (74.3)	66 (68.0)	0.294 ^b^
	Yes	67 (28.3)	26 (31.7)	41 (26.5)		36 (25.7)	31 (31.0)	
F4. Food price	No	122 (51.5)	44 (53.7)	78 (50.3)	0.625 ^b^	72 (51.4)	50 (51.5)	0.986 ^b^
	Yes	115 (48.5)	38 (46.3)	77 (49.7)		68 (48.6)	47 (48.5)	
F5. Managing weight	No	227 (95.8)	76 (92.7)	151 (97.4)	0.099 ^a^	134 (95.7)	93 (95.9)	1.000 ^a^
	Yes	10 (4.2)	6 (7.3)	4 (2.6)		6 (4.3)	4 (4.1)	
F6. Food availability	No	210 (88.6)	71 (86.6)	139 (89.7)	0.476 ^b^	122 (87.1)	88 (90.7)	0.394 ^b^
	Yes	27 (11.4)	11 (13.4)	16 (10.3)		18 (12.9)	9 (9.3)	
F7. Presentation or packaging	No	231 (97.5)	80 (97.6)	151 (97.4)	1.000 ^a^	137 (97.9)	94 (96.9)	0.691 ^a^
	Yes	6 (2.5)	2 (2.4)	4 (2.6)		3 (2.1)	3 (3.1)	
F8. Another person decides most of the foods that I eat	No	233 (98.3)	81 (98.8)	152 (98.1)	1.000 ^a^	137 (97.9)	96 (99.0)	0.646 ^a^
	Yes	4 (1.7)	1 (1.2)	3 (1.9)		3 (2.1)	1 (1.0)	
F9. Vegetarian food or other special habits	No	234 (98.7)	82 (100.0)	152 (98.1)	0.553 ^a^	138 (98.6)	96 (99.1)	1.000 ^a^
	Yes	3 (1.3)	0 (0.0)	3 (1.9)		2 (1.4)	1 (1.0)	
F10. Content of additives, dyes, and preservatives	No	232 (97.9)	79 (96.3)	153 (98.7)	0.344 ^a^	136 (97.1)	96 (99.1)	0.651 ^a^
	Yes	5 (2.1)	3 (3.7)	2 (1.3)		4 (2.9)	1 (1.0)	
F11. My cultural, religious, or ethnic roots	No	234 (98.7)	80 (97.6)	154 (99.4)	0.275 ^a^	137 (97.9)	97 (100.0)	0.272 ^a^
	Yes	3 (1.3)	2 (2.4)	1 (0.6)		3 (2.1)	0 (0.0)	
F12. Ease or convenience of preparation	No	95 (40.1)	34 (41.5)	61 (39.4)	0.753 ^b^	54 (38.6)	41 (42.3)	0.568 ^b^
	Yes	142 (59.9)	48 (58.5)	94 (60.6)		86 (61.4)	56 (57.7)	
F13. Trying to have a healthy diet	No	222 (93.7)	77 (93.9)	145 (93.5)	0.915 ^b^	131 (93.6)	91 (93.8)	0.940 ^b^
	Yes	15 (6.3)	5 (6.1)	10 (6.5)		9 (6.4)	6 (6.2)	
F14. Quality or freshness of food	No	222 (93.7)	76 (92.7)	146 (94.2)	0.650 ^b^	129 (92.1)	93 (95.9)	0.246 ^b^
	Yes	15 (6.3)	6 (7.3)	9 (5.8)		11 (7.9)	4 (4.1)	
F15. Other—Aspects related to the establishment/ service (customer service, environment)	No	230 (97.0)	81 (98.8)	149 (96.1)	0.427 ^a^	135 (96.4)	95 (97.9)	0.703 ^a^
	Yes	7 (3.0)	1 (1.2)	6 (3.9)		5 (3.6)	2 (2.1)	
F16. Other—Socializing with friends/family	No	223 (94.1)	79 (96.3)	144 (92.9)	0.390 ^a^	136 (97.1)	87 (89.7)	0.017 *
	Yes	14 (5.9)	3 (3.7)	11 (7.1)		4 (2.9)	10 (10.3)	
F17. Other—Lack of desire to cook	No	232 (97.9)	81 (98.8)	151 (97.4)	0.663 ^a^	136 (97.1)	96 (99.0)	0.651 ^a^
	Yes	5 (2.1)	1 (1.2)	4 (2.6)		4 (2.9)	1 (1.0)	
F18. Other—Quantity/ size/portion of meal	No	236 (99.6)	82 (100.0)	154 (99.4)	1.000 ^a^	139 (99.3)	97 (100.0)	1.000 ^a^
	Yes	1 (0.4)	0 (0.0)	1 (0.6)		1 (0.7)	0 (0.0)	
F19. Other—Craving or desire for fast food	No	204 (86.1)	77 (93.9)	127 (81.9)	0.011 *^b^	120 (85.7)	84 (86.6)	0.847 ^b^
	Yes	33 (13.9)	5 (6.1)	28 (18.1)		20 (14.4)	13 (13.4)	
**Factors That Have the Greatest Influence on Your Choice for Fast Food:**	**No/** **Yes**	**Do You Usually Consume Fast Food?**			**Body Mass Index (kg/m^2^)**		**Amount Spent on Each Fast Food Meal (EUR)**	
		**No** **n (%)**	**Yes** **n (%)**	***p***	**Mean (SD)**	***p* ^c^**	**Mean (SD)**	***p* ^c^**
F1	No	54 (94)	178 (98.3)	0.338 ^a^	22.9 (3.5)	0.734	8.6 (2.9)	0.045 *
	Yes	2 (3.6)	3 (1.7)		23.1 (2.7)		11.2 (2.9)	
F2	No	45 (80.4)	155 (85.6)	0.342 ^b^	22.9 (3.4)	0.470	8.5 (3.0)	0.091
	Yes	11 (19.6)	26 (14.4)		23.4 (3.8)		9.3 (2.9)	
F3	No	42 (75.0)	128 (70.7)	0.534 ^b^	23.0 (3.7)	0.571	8.7 (3.0)	0.508
	Yes	14 (25.0)	53 (29.3)		23.0 (2.9)		8.4 (2.8)	
F4	No	28 (50.0)	94 (51.9)	0.800 ^b^	23.1 (3.2)	0.549	9.4 (3.3)	<0.001 *
	Yes	28 (50.0)	87 (48.1)		22.9 (3.8)		7.8 (2.2)	
F5	No	49 (87.5)	178 (98.3)	0.002 *^a^	22.8 (3.3)	0.006*	8.6 (3.0)	0.221
	Yes	7 (12.5)	3 (1.7)		26.2 (4.4)		9.5 (2.5)	
F6	No	48 (85.7)	162 (89.5)	0.436	22.9 (3.1)	0.847	8.8 (2.9)	0.038 *
	Yes	8 (14.3)	19 (10.5)		23.8 (5.7)		7.7 (3.3)	
F7	No	55 (98.2)	176 (97.2)	1.000 ^a^	23.0 (3.5)	0.619	8.7 (3.0)	0.639
	Yes	1 (1.8)	5 (2.8)		22.5 (3.6)		7.8 (1.7)	
F8	No	55 (98.2)	178 (98.3)	1.000 ^a^	23.0 (3.5)	0.362	8.6 (3.0)	0.184
	Yes	1 (1.8)	3 (1.7)		21.3 (2.7)		10.6 (3.5)	
F9	No	55 (98.2)	179 (98.9)	0.556 ^a^	23.0 (3.5)	0.426	8.6 (3.0)	0.831
	Yes	1 (1.8)	2 (1.1)		23.7 (1.9)		8.2 (2.5)	
F10	No	54 (96.4)	178 (98.3)	0.338 ^a^	23.0 (3.5)	0.512	8.6 (3.0)	0.622
	Yes	2 (3.6)	3 (1.7)		24.1 (4.4)		8.5 (0.9)	
F11	No	55 (98.2)	179 (98.9)	0.556 ^a^	23.0 (3.5)	0.729	8.6 (3.0)	0.935
	Yes	1 (1.8)	2 (1.1)		22.0 (2.3)		8.5 (3.1)	
F12	No	24 (42.9)	71 (39.2)	0.628 ^b^	23.0 (3.2)	0.582	8.8 (3.0)	0.733
	Yes	32 (57.1)	110 (60.8)		23.0 (3.7)		8.5 (3.0)	
F13	No	48 (85.7)	174 (96.1)	0.010 *^a^	23.0 (3.2)	0.761	8.8 (3.0)	0.095
	Yes	8 (14.3)	7 (3.9)		23.8 (4.8)		9.7 (2.7)	
F14	No	50 (89.3)	172 (95.0)	0.128 ^a^	23.0 (3.5)	0.392	8.6 (3.0)	0.953
	Yes	6 (10.7)	9 (5.0)		22.2 (2.5)		8.6 (2.9)	
F15	No	56 (100.0)	174 (96.1)	0.203 ^a^	23.0 (3.5)	0.782	8.7 (3.0)	0.331
	Yes	0 (0.0)	7 (3.9)		23.0 (3.1)		7.6 (1.5)	
F16	No	54 (96.4)	169 (93.4)	0.529 ^a^	22.9 (3.5)	0.320	8.7 (3.0)	0.277
	Yes	2 (3.6)	12 (6.6)		23.0 (3.1)		7.4 (1.5)	
F17	No	56 (100.0)	176 (97.2)	0.594 ^a^	23.0 (3.5)	0.365	8.7 (3.0)	0.285
	Yes	0 (0.0)	5 (2.8)		23.5 (2.1)		7.4 (1.5)	
F18	No	56 (100.0)	180 (99.4)	1.000 ^a^	23.0 (3.5)	0.312	8.7 (3.0)	0.118
	Yes	0 (0.0)	1 (0.6)		19.9 (-.-)		5.0 (-.-)	
F19	No	52 (92.9)	152 (84.0)	0.093 ^b^	23.2 (3.6)	0.001 *	8.6 (3.0)	0.499
	Yes	4 (7.1)	29 (16.0)		21.5 (2.6)		8.9 (3.1)	

* p < 0.005; ^a^ Fisher’s exact test; ^b^ Chi-squared test; ^c^ Mann–Whitney test.

## Data Availability

Data are contained within the article.
